# Haemostatic changes during CART cell therapy and risk of complications

**DOI:** 10.1007/s00262-026-04371-6

**Published:** 2026-03-31

**Authors:** María Panizo-Inogés, María Marcos-Jubilar, Jose Ramón González-Porras, Carlos Puerta-Vazquez, Clara Fernández-Arias, Paula Rodríguez-Otero, Ana Alfonso-Pierola, Sara Villar, Miguel Ángel Canales, Josune Orbe, Jose Antonio Páramo, Felipe Prósper, Ramón Lecumberri

**Affiliations:** 1 Hematology Department, Clinica Universidad de Navarra, Pamplona, Spain; 2 Hematology Department, Hospital Universitario de Salamanca, Salamanca, Spain

**Keywords:** Bleeding, CART cell therapy, P-selectin, Thrombosis, Thrombin generation

## Abstract

Chimeric antigen receptor T cell (CART) therapy toxicity includes cytokine release syndrome (CRS), immune effector cell-associated neurotoxicity syndrome (ICANS), but also thrombotic and haemorrhagic events. Given the link between haemostasis and inflammation, haemostatic biomarkers could help identify at-risk patients. Sixty-two adult patients receiving CD19- or BCMA-targeted CART were prospectively included and followed up for 30 days to characterize haemostatic changes and evaluate their association with the risk of CRS, ICANS, thrombosis and bleeding. Blood samples were collected at baseline (pre-lymphodepletion), pre-infusion and on days + 3, + 14, and + 28 post-infusion. Haemostatic tests, including thrombin generation assay (TGA, ST-Genesia) and P-Selectin, were performed. CRS occurred in 94% of patients, ICANS in 39%, venous thrombosis in 1.6%, and clinically relevant bleeding in 13%. During follow-up, prolonged coagulation times, thrombocytopenia and decreased fibrinogen and P-selectin, were noted. Baseline endogenous thrombin potential (ETP) was associated with clinically relevant CRS risk (OR 12.39; *p* = 0.02) and baseline C-reactive protein (CRP) with ICANS (OR 1.66; *p* < 0.01). Baseline P-selectin levels and platelet count identified patients at higher bleeding risk (OR 0.20; *p* = 0.03 and OR 0.39; *p* < 0.01, respectively). In the first month after CART infusion, bleeding incidence exceeds thrombosis. TGA, CRP, platelet count and P-selectin may help identify patients at risk of severe toxicity, enabling earlier, targeted interventions.

**Supplementary Information:**

The online version contains supplementary material available at 10.1007/s00262-026-04371-6.

## Introduction

Chimeric antigen receptor T cell (CART) therapy has revolutionized the treatment of various haematological malignancies, offering an effective option for patients with relapsed or refractory disease [1–7]. However, CART therapies are associated with a unique toxicity profile, including cytokine release syndrome (CRS), immune effector cell-associated neurotoxicity syndrome (ICANS) and immune effector cell-associated hematotoxicity (ICAHT) [8–12]. Thrombotic and haemorrhagic complications have also been reported, highlighting the link between inflammation and haemostasis.

As CART therapies gain broader use, progress has been made in understanding the pathophysiology of CRS, ICANS, and ICAHT [12, 13]. However, haemostatic complications, including thrombosis and bleeding, remain less well characterized. Identifying patients at high risk for these events remains a major clinical challenge.

Reported rates of thrombotic and haemorrhagic events following CART therapy vary considerably. A recent meta-analysis showed that the risk is higher during the first months following infusion. Overall, the incidence of venous thrombotic events was 2.4% per patient-month, while the incidence of clinically relevant haemorrhagic events was 1.9% per patient-month, underscoring the need for active monitoring during the early post-infusion period [14].

The thrombin generation assay (TGA) is a global coagulation test that captures the dynamic interplay between procoagulant and anticoagulant forces. Unlike conventional static tests such as prothrombin time (PT) and activated partial thromboplastin time (aPTT), TGA offers a comprehensive evaluation of the coagulation status. It has been applied to identify hypo- or hypercoagulable states and to assess bleeding and thrombotic risks in diverse settings [15–19]. P-selectin, a cell adhesion protein expressed on activated endothelial cells and platelets, plays an important role in leukocyte recruitment during inflammation. Elevated plasma levels have been associated with thrombotic risk [20–23].

We hypothesized that thrombin generation assessment, together with other markers of coagulation or endothelium activation, could aid in risk stratification and prediction of inflammatory and haemostatic complications in CART recipients. This study aimed to characterize the dynamics of haemostatic alterations in patients undergoing CART therapy and assess their association with inflammation and thrombotic or haemorrhagic risk.

## Patients and methods

### Patients and study design

Consecutive adult patients with haematologic malignancies scheduled for CART therapy at Clínica Universidad de Navarra or Hospital Universitario de Salamanca were prospectively recruited. Patients requiring therapeutic anticoagulation were excluded. Blood samples were collected at five time points: before lymphodepletion (baseline), pre-infusion, and on days + 3 (CRS was expected to develop at this time point in most patients), + 14, and + 28 post-infusion. The study was approved by the local ethics committee and conducted in accordance with the Declaration of Helsinki. All participants provided written informed consent prior to inclusion.

### Laboratory analysis

Venous blood samples were obtained in 3.2% sodium citrate tubes (9:1 ratio) (Vacutainer, Becton Dickinson, Franklin Lakes, NJ, USA) through sterile, atraumatic venipuncture. PT, aPTT, fibrinogen, D-dimer and Von Willebrand factor (vWF) were processed following routine protocols using ACL-TOP 350 or STA-Compact analysers.

Blood samples for TGA and P-selectin assay were double-centrifuged (1500 g for 10 min to separate the plasma, then 2500 g for 15 min) to obtain platelet-poor plasma. Aliquots (400 μL) were stored at −80 °C and thawed at 37 °C for 5 min before analysis.

Thrombin generation was measured using a commercially available assay kit in the presence or absence of thrombomodulin (TM) (Thromboscreen kit, ST-Genesia, Stago), that includes coagulation activators with and without thrombomodulin, reference plasma for normalization and quality controls. This technique was performed on ST-Genesia fully automated analyser (Diagnostica Stago, Asnières-sur-Seine, France), following manufacturers recommendations, including daily calibration curves. Parameters evaluated were lag time, time to peak, thrombin peak height, endogenous thrombin potential (ETP), and ETP inhibition by TM (ETPinh) (Suppl. Figure 1). Normalized values were used, following ISTH recommendations [24]. P-selectin was determined using Quantikine Human P-Selectin/CD62P Immunoassay (R&D Systems) and C-reactive protein (CRP) using Tina-quant C-Reactive Protein IV (Roche Diagnostics).

### Clinical outcomes

Patients were followed for 30 days post-infusion. Outcomes of interest (CRS, ICANS, thrombosis and bleeding) were assessed and adjudicated by the study team. Assessment was performed daily until discharge (which in all cases occurred beyond day + 14 after CART infusion). After discharge and until day + 30, clinical assessment was performed at least once weekly. Symptomatic thrombotic events were confirmed via imaging, including compression ultrasonography or CT scan.

Haemorrhagic event severity was graded per ISTH criteria [25, 26], while CRS and ICANS were graded according to the ASTCT Consensus Grading for Cytokine Release Syndrome and Neurologic Toxicity Associated with Immune Effector Cells [27]. CRS grade ≥ 2 and any grade ICANS were considered clinically relevant. Endothelial dysfunction was estimated using the EASIX score, calculated as LDH (U/L) × creatinine (mg/dL)/platelets (10^9^/L) [28, 29]. The CAR-HEMATOTOX score was determined prior to lymphodepletion. One point was assigned for: absolute neutrophil count ≤ 1200/μl, haemoglobin ≤ 9.0 g/dl, platelet count 75–175 × 10⁹/L, C-reactive protein ≥ 3.0 mg/dl, and ferritin 650–2000 ng/ml. Two points were assigned for platelet count ≤ 75 × 10⁹/L or ferritin ≥ 2000 ng/ml. Patients scoring ≥ 2 were categorized as high risk; those with 0–1, as low risk [30, 31].

To evaluate the associations between analytical variables and outcomes, baseline samples and those obtained at the time points immediately before the occurrence of each event were used.

### Statistical analysis

Continuous variables were described by mean ± standard deviation (SD) or median and interquartile range (IQR), depending on distributions. Nominal variables were described as counts and percentages. For variables with clinically relevant cut-offs (e.g. vWF activity/vWF antigen ratio < 0.7), dichotomization was applied.

Fisher’s exact test was applied for categorical comparisons. Continuous biomarkers were log₂-transformed to normalize their distribution; thus, estimated coefficients represent the effect associated with a doubling of the biomarker level. Univariable logistic regression assessed associations, expressed as odds ratios (OR) and 95% confidence intervals (CI). Variables with p < 0.05 were included in multivariable logistic regression. Cut-off points were determined using receiver operating characteristic (ROC) curve analysis, with the Youden index used to define optimal thresholds. The ROC/AUC was calculated using the original scale of the biomarker, since AUC is unaffected by monotonic transformations.

All analyses were performed using StataCorp LP 2012, with p < 0.05 considered statistically significant. A complete-case approach was used for all analyses; missing data were excluded from the corresponding analyses, without imputation.

## Results

### Patients characteristics

Between February 2022 and July 2024, 62 patients, 40 men and 22 women (median age 62 years [IQR 53–69 years]), were included. Baseline characteristics are shown in Table 1. The cohort included 31 patients (50%) with B cell lymphomas (BCL), 29 patients (47%) with multiple myeloma (MM), and 2 patients (3%) with B cell acute lymphoblastic leukaemia (B-ALL). The median number of prior treatment lines before CART therapy was 2 (IQR 2–3). At infusion, 94% (n = 58) of patients had an ECOG performance status of 0–1. Disease status at this time was categorized as no response in 41 patients (66%), partial response in 14 patients (23%) and complete response in 7 patients (11%).

**Table 1:** Baseline characteristics of the recruited patients

	Total (n = 62)	Anti-CD19 CART (n = 33)	Anti-BCMA CART (n = 29)	*p* Value
Age (years) (median, IQR)	62 (53−69)	62 (53−68)	65 (53−69)	0.54
Sex, n (%)				
Male Female	40 (65) 22 (35)	23 (70) 10 (30)	17 (59) 12 (41)	0.36
Diagnosis, n (%)				
B-ALL B cell lymphomas Multiple myeloma	2 (3) 31 (50) 29 (47)	2 (6) 31 (94) NA	NA NA 29 (100)	NA
Disease status pre-lymphodepletion, n (%)				
CR PR NR/PD	7 (11) 14 (23) 41 (66)	2 (6) 5 (15) 26 (79)	5 (17) 9 (31) 15 (52)	0.07
History of venous thrombosis, n (%) PE ± DVT Isolated lower-limb DVT CRT	15 (24) 7 (12) 4 (6) 4 (6)	8 (24) 2 (6) 4 (12) 2 (6)	7 (24) 5 (17) 0 (0) 2 (7)	0.99
History of arterial thrombosis, n (%) Myocardial infarction Stroke	5 (8) 2 (3) 3 (5)	4 (12) 2 (6) 2 (6)	1 (3) 0 (0) 1 (3)	0.61
Cardiovascular risk factors, n (%)				
HT DM DL CKD	20 (32) 10 (16) 16 (25) 7 (7)	11 (33) 9 (27) 10 (30) 6 (18)	9 (31) 1 (3) 6 (21) 1 (3)	0.85 0.01 0.39 0.11
Thromboprophylaxis upon admission, n (%) Duration (days) (median, IQR)	51 (82) 14 (12−15)	25 (75) 14 (10−18)	26 (90) 14 (13−14)	0.22
CAR-HEMATOTOX ≥ 2 points, n (%)	24 (39)	16 (48)	8 (28)	0.07
EASIX score (median, IQR)	1.21 (0.78−2.59)	1.52 (1.03−2.59)	1.04 (0.69−2.47)	0.07

B-ALL: B cell acute lymphoblastic leukaemia. CR: complete response. PR: partial response. NR: no response. PD: progressive disease. PE ± DVT: pulmonary embolism ± deep vein thrombosis. CRT: catheter-related thrombosis. HT: hypertension. DM: diabetes mellitus. DL: dyslipidaemia. CKD: chronic kidney disease. IQR: interquartile range. NA: Not applicable

Fifteen patients (24%) had a previous history of venous thromboembolism (VTE): 7 pulmonary embolisms (PE) ± deep vein thrombosis (DVT), 4 isolated lower-limb DVT and 4 upper-limb catheter-related thrombosis (CRT). The median time from VTE to CART infusion was 10 months (IQR 7.5–40 months). Additionally, 5 patients (8%) had a history of arterial thrombosis: 2 myocardial infarctions and 3 ischaemic strokes, with a median time to CART infusion of 120 months (IQR 108–144 months). No major haemorrhages were documented in the month prior to infusion. Median baseline EASIX score was 1.21 (IQR 0.78–2.59) and 39% of patients had a high-risk CAR-HEMATOTOX score (≥ 2 points). No relevant differences were observed between CD19 and BCMA recipients (Table 1).

All patients received lymphodepletion with cyclophosphamide and fludarabine for 3 days, starting 5 days before infusion. Cellular products, targeting CD19 or BCMA, were infused. Following institutional protocols, during hospitalization, 51 patients (82%) received LMWH thromboprophylaxis, which was discontinued whenever the platelet count dropped < 50 × 10^9^/L. The median duration of thromboprophylaxis was 14 days (IQR 12–15 days). Four patients (6%) maintained LMWH prophylaxis throughout the whole study period.

### Clinical outcomes during follow-up

**Fig. 1:**
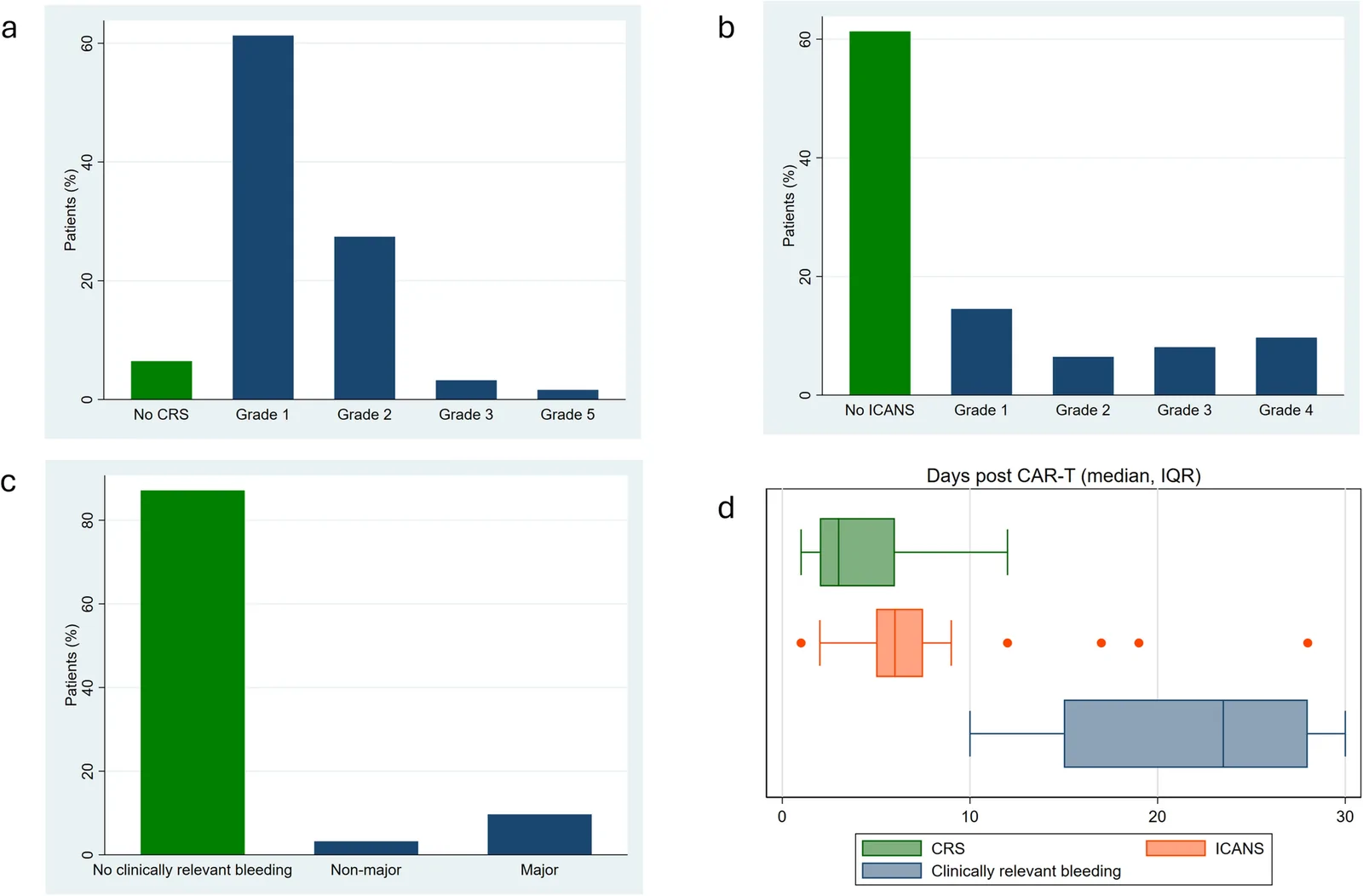
Distribution and onset of key complications during follow-up. **a** Distribution of CRS grades. **b** Distribution of ICANS grades. **c** Distribution of haemorrhage grades. **d** Median time to onset of each complication after CART cell infusion

Overall, 58 patients (94%) experienced CRS: 38 grade 1, 17 grade 2, and 3 grade ≥ 3, including one grade 5 CRS. All cases occurred early, with a median onset of 3 days post-infusion (IQR 2–6) (Fig. 1). Tocilizumab, alone or combined with dexamethasone or anakinra, was required in 49 patients (84%), 30 of them due to grade 1 CRS with persistent fever.

ICANS was recorded in 24 patients (39%): 9 grade 1, 4 grade 2, and 11 grade ≥ 3. Again, onset was relatively early, with a median of 6 days post-infusion (IQR 5–7) (Fig. 1). All ICANS cases were preceded by CRS, with 11 (46%) having clinically relevant CRS (defined as grade ≥ 2).

During follow-up, 8 patients (13%), 6 with BCL and 2 with MM, experienced a major or clinically relevant haemorrhagic event. Locations included gastrointestinal (n = 4), central nervous system (n = 2), urinary (n = 1) and soft tissues (n = 1). Bleeding events occurred relatively late, with a median onset of 24 days after CART infusion (IQR 14–28) (Fig. 1), all but one occurring from day 14 onwards. Only one patient was on LMWH prophylaxis at bleeding onset (Suppl. Table 1). Interestingly, all patients who suffered a major or clinically relevant bleeding event had prior CRS (5 grade 1, 3 grade 2) and 6 patients had developed ICANS. Clinically relevant CRS was not significantly associated with bleeding (OR: 1.3, 95% CI: 0.3–6.1; *p* = 0.7), but ICANS was (OR: 6.0, 95% CI: 1.1–32.8; *p* = 0.03).

Only 1 (1.6%) VTE event (CRT) was observed within the first month post-infusion, on day + 20 in a BCL patient not receiving thromboprophylaxis at that time. No arterial thrombotic complications were observed during follow-up. Three patients died on days + 11, + 26, and + 27 after infusion due to CRS, disease progression, and haemorrhage, respectively.

### Haemostatic tests

The dynamics of laboratory assessments across the different study time points is shown in Table 2 and Supplementary Fig. 2. After lymphodepletion, a transient delay in thrombin generation was observed. Lag time and time-to-peak ratios significantly increased at infusion day (p < 0.05 vs. baseline) and remained prolonged until day + 3, gradually normalizing by day + 14. In parallel, peak height decreased significantly pre-infusion and at day + 3 (p < 0.05), recovering thereafter. However, ETP and ETP inhibition remained largely stable throughout follow-up, showing no significant fluctuations.

**Table 2:** Dynamics of thrombin generation variables and other haemostatic tests

		Baseline	Pre-infusion	Day + 3	Day + 14	Day + 28
Thrombin generation	Lag time (ratio)	1.3 (1.1–1.5)	1.4 (1.2–1.9) *	1.3 (1.2–1.8) *	1.2 (1.0–1.5) †	1.2 (1–1.5) †
	Peak Height (%)	107.3 (73.1–137.0)	80.6 (57.2–108.4) *	90.5 (61.5–118.3) *	97.5 (75.3–122.4) †	91.1 (67.8–115.7) †
	Time to Peak (ratio)	1.2 (0.9–1.4)	1.3 (1.1–1.6) *	1.3 (1.1–1.6) *	1.2 (1.1–1.4) †	1.2 (1.1–1.6) †
	ETP (%)	99.3 (78.9–106.5)	92.7 (72.4–109.1)	97.4 (80.3–111.1)	94.1 (81.5–107.7)	94.8 (77.5–106.6)
	TM ETP Inh (%)	47.4 (26.7–62.2)	51.1 (39.1–64.6)	48.6 (32.8–57.1)	50.1 (37.6–61.7)	52.1 (33.0–72.9)
Prothrombin Time (s) (RV: 9.2–13.8)		9.7 (8.4–13.9)	12.2 (8.2–14.1)	11.5 (8.4–14.6) * †	11.2 (9.3–14.4) * †	11.4 (8.9–13.4)*†
Activated Partial Thromboplastin Time (s) (RV: 24.5–35.5)		30.5 (27.9–33.2)	31.1 (28.2–34.6)	33.8 (28.5–37.4) * †	27.5 (24.3–31.3) * †	26.6 (24.2–30.3)* †
D-dimer (ng/mL) (RV: < 500)		570 (300–930)	700 (440–1500) *	900 (500–1500) *	900 (570–1940) * †	702 (400–1455)
Fibrinogen (mg/dL) (RV: 200–393)		390 (328–478)	409 (344–463)	415 (360–473)	182 (142–227) * †	182 (152–242)*†
vWF Act/Ag (ratio) (RV ≥ 0.7)		0.9 (0.8–1.1	0.9 (0.8–1.1)	0.9 (0.8–1.1)	0.8 (0.7–0.9) * †	0.8 (0.6–0.9) *†
CRP (mg/dL) (RV ≤ 0.5)		0.6 (0.1–1.8)	0.7 (0.4–2.3)	2.2 (0.5–5.9) * †	0.2 (0.1–0.4) * †	0.1 (0.04–0.1)*†
Platelets (× 10^9^/L) (RV: 150–450)		156 (106–201)	125 (66–161) *	101 (51–145) * †	101 (31–152) * †	65 (16–112) * †
P-selectin (ng/ml)		46.4 (38.8–55.3)	31.6 (25.9–38.4)*	30.0 (23.3- 39.8)* †	40.5 (27.7–50.3)* †	NA

Available reference values are provided where applicable. All data are presented as median (interquartile range). * p < 0.05 compared to pre-lymphodepletion. † p < 0.05 compared to pre-infusion. ETP: endogenous thrombin potential. TM: thrombomodulin. vWF Act/Ag: von Willebrand factor activity/antigen ratio. CRP: C-reactive protein. RV: reference values. NA: not available. At day + 28, 18 cases were missing due to losses to follow-up.

**Fig. 2:**
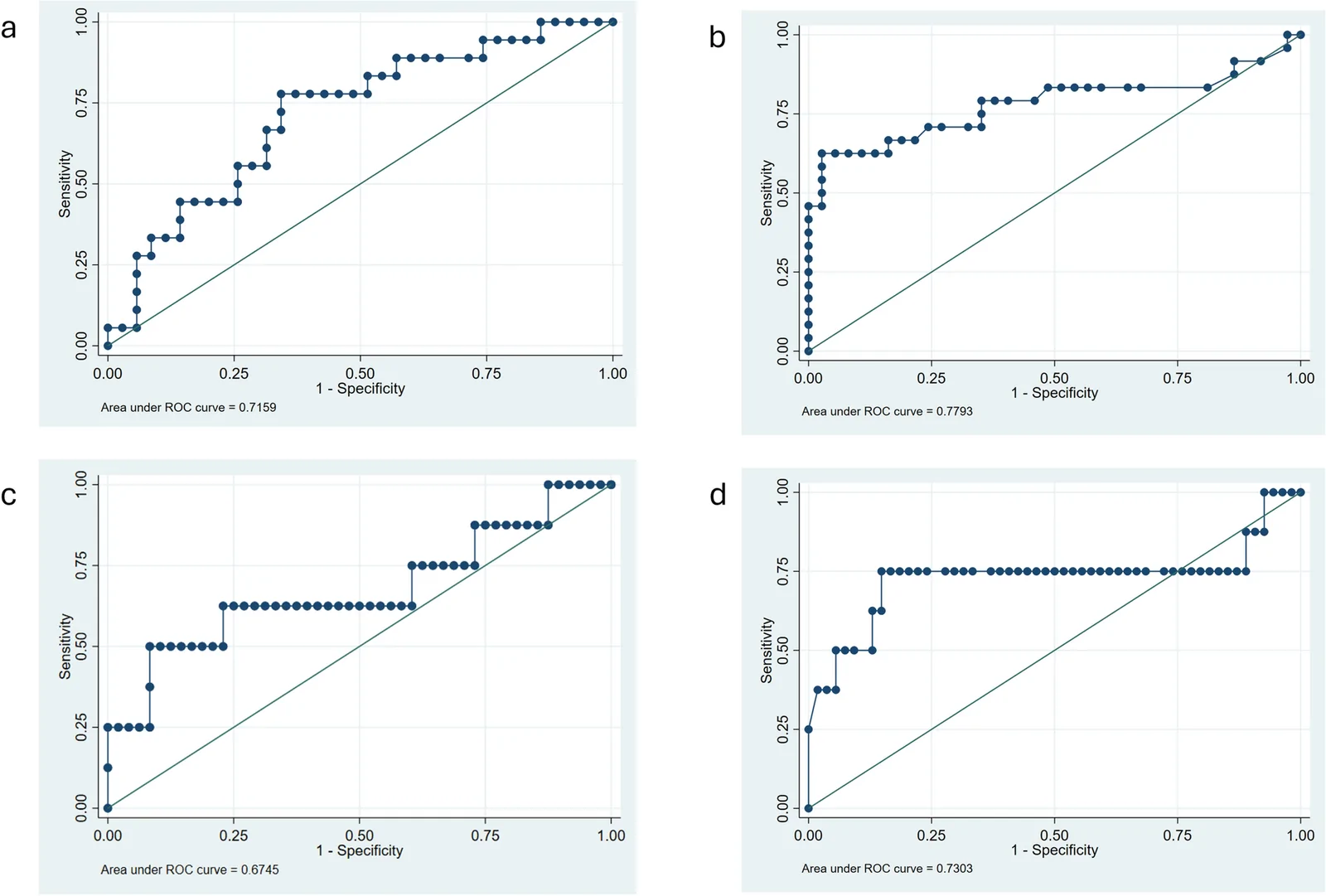
ROC curves evaluating the predictive performance of biomarkers for study outcomes **a** ROC curve of baseline ETP in patients with clinically relevant CRS. **b** ROC curve of baseline CRP in patients with ICANS. **c** ROC curve of baseline P-selectin in patients with clinically relevant bleeding. **d** ROC curve of baseline platelet count in patients with clinically relevant bleeding

Regarding conventional coagulation tests, PT and APTT were significantly prolonged at day + 3, compared to baseline values. While the prolongation of PT persisted thereafter, the APTT significantly shortened by days + 14 and + 28. Fibrinogen levels remained stable until day + 3 but dropped significantly afterwards. On the contrary, D-dimer levels rose significantly from baseline to pre-infusion and remained elevated until day + 14 (p < 0.05), before showing partial normalization by day + 28.

CRP concentrations peaked markedly at day + 3 (median 2.2 mg/dL, p < 0.05 vs. both baseline and pre-infusion), followed by a significant reduction at days + 14 and + 28, where values fell below baseline and pre-infusion levels (p < 0.05 vs. both time points).

Endothelial and platelet activation markers also varied significantly over time. A delayed (days + 14 and + 28) vWF activity/antigen ratio decrease was observed. Circulating P-selectin concentrations decreased markedly after lymphodepletion (p < 0.05), with partial recovery by day + 14. Of note, platelet counts progressively decreased from baseline through day + 28, with statistically significant reductions at all post-baseline assessments (p < 0.05).

### Haemostatic changes and risk of clinical outcomes

### Clinically relevant CRS

Univariable logistic regression showed that baseline ETP and peak height were significantly associated with clinically relevant CRS (OR 12.39, 95% CI 1.46–105.06; *p* = 0.02 and OR 4.50, 95% CI 1.33–15.26; *p* = 0.02, respectively) (Table 3). The ETP AUC was 0.72 (95% CI 0.6–0.9), with an optimal cut-off of 99.8% (sensitivity 77.8%, specificity 65.7%) (Fig. 2a).

**Table 3:** Association of haemostatic biomarker with clinical outcomes (univariable logistic regression analysis)

	Clinically relevant CRS	ICANS		Clinically relevant bleeding	
	Baseline OR (95% CI)	Baseline OR (95% CI)	Day + 3 OR (95% CI)	Baseline OR (95% CI)	Day + 3 OR (95% CI)
ETP (%)	12.39 (1.46–105.06)*	0.42 (0.09–1.89)	0.47 (0.13–1.66)	0.68 (0.09–5.11)	0.35 (0.06–2.14)
Lag time (ratio)	0.37 (0.11–1.24)	3.85 (1.21–12.28)*	5.69 (1.51–21.41)*	3.24 (0.90–11.67)	4.26 (0.86–21.17)
Peak height (%)	4.50 (1.33–15.26)*	0.66 (0.27–1.62)	0.44 (0.18–1.05)	0.58 (0.17–1.96)	0.54 (0.16–1.78)
Time to peak (ratio)	0.20 (0.04–1.02)	3.02 (0.85–10.74)	8.39 (1.67–42.16)*	2.73 (0.60–12.35)	3.50 (0.48–25.20)
TM ETP Inh (%)	0.52 (0.25–1.10)	1.49 (0.70–3.17)	2.61 (0.93–7.31)	2.33 (0.64–8.44)	1.36 (0.36–5.11)
Prothrombin time (seg)	0.87 (0.20–3.85)	0.54 (0.14–2.17)	0.30 (0.08–1.11)	2.51 (0.36–17.49)	0.62 (0.09–4.15)
Activated Partial Thromboplastin time (seg)	0.55 (0.03–9.49)	2.48 (0.18–35.11)	1.12 (0.18–6.77)	52.52 (0.83–3310.00)	1.32 (0.10–17.65)
D-dimer (ng/mL)	1.10 (0.66–1.84)	1.71 (0.99–2.95)	1.46 (0.93–2.30)	1.02 (0.47–2.22)	1.14 (0.63–2.07)
Fibrinogen (md/dL)	0.74 (0.25–2.15)	1.82 (0.57–5.78)	2.34 (0.62–8.91)	4.41 (0.53–36.89)	1.35 (0.23–7.83)
vWF Act/Ag < 0.7 (ratio)	0.72 (0.16–3.20)	2.8 (0.68–11.49)	3.88 (0.86–17.48)	3.26 (0.63–16.84)	5.75 (1.03–32.17)*
CRP (mg/dL)	1.14 (0.93–1.41)	1.66 (1.26–2.19)†	1.49 (1.11–1.99)†	1.37 (1.01–1.87)*	1.20 (0.82–1.76)
Platelets (× 10^9^/L)	0.86 (0.54–1.37)	0.71 (0.44–1.14)	0.69 (0.47–1.00)	0.39 (0.21–0.74)†	0.53 (0.33–0.88)*
P-selectin (ng/ml)	0.83 (0.32–2.15)	1.01 (0.39–2.60)	0.61 (0.29–1.32)	0.20 (0.05–0.82)*	0.37 (0.13–1.04)

*p < 0.05; † p < 0.01. ETP: endogenous thrombin potential. TM: thrombomodulin. vWF Act/Ag: von Willebrand factor activity/antigen ratio. CRP: C-reactive protein

No other baseline or pre-infusion parameters were significantly associated with clinically relevant CRS (Table 3).

### ICANS

At baseline, univariable logistic regression showed that TGA lag time and CRP were significantly associated with the development of ICANS (Table 3). In the multivariable model, only CRP retained a significant association with ICANS (OR 1.86, 95% CI 1.28–2.69; *p* < 0.01). The CRP AUC was 0.78 (95% CI 0.6–0.9) with an optimal cut-off of 1.66 mg/dL (sensitivity 62.5%, specificity 94.6%) (Fig. 2b).

The univariable analysis conducted at day + 3, as the closest point prior to the events, revealed that CRP, TGA lag time and time-to-peak significantly associated with the risk of ICANS. Indeed, both TGA parameters were highly correlated (R^2^ = 0.80). In the multivariable analysis none of them remained statistically significant, although CRP approached significance (*p* = 0.05).

### Clinically relevant bleeding

At baseline, univariable logistic regression showed that each doubling of P-selectin levels was associated with an 80% reduction in the odds of bleeding (OR 0.20, 95% CI 0.05–0.82; *p* = 0.03), while each doubling of platelet count reduced the odds by 61% (OR 0.39, 95% CI 0.21–0.74; *p* < 0.01). Additionally, each doubling of CRP levels increased the odds of bleeding 1.37-fold (OR 1.37, 95% CI 1.01–1.87; *p* = 0.04) (Table 3). The low number of events did not allow to perform a multivariable analysis including all these variables. However, in bivariable analysis platelet count and P-selectin remained significantly associated with bleeding. The P-selectin AUC was 0.67 (95% CI 0.42–0.93) with an optimal cut-off of 34.8 ng/ml (sensitivity 50.0%, specificity 89.6%) (Fig. 2c). The platelet count AUC was 0.73 (95% CI 0.45–1.0) with an optimal cut-off of 102 × 10^9^/L (sensitivity 75.0%, specificity 83.3%) (Fig. 2d).

Median baseline EASIX score was 6.0 in patients who suffered a bleeding event during follow-up vs. 1.1 in those who did not bleed (p < 0.01). A high-risk CAR-HEMATOTOX score was not associated with bleeding risk (OR 2.8, 95% CI 0.6–13.1; *p* = 0.19).

On day + 3, univariable analysis showed that platelet count and vWF ratio < 0.7 were significantly associated with bleeding (OR 0.53, 95% CI 0.33–0.88; *p* = 0.01 and or 5.75, 95% CI 1.03–32.17; *p* = 0.04, respectively) (Table 3). When both variables were included in a bivariable model, only the platelet count remained significantly associated (OR 0.50, 95% CI 0.28–0.89; *p* = 0.02).

As all but one bleeding event occurred after day + 14, a sensitivity analysis was performed at this time point, excluding the day + 10 event. At day + 14, lower ETP (OR 0.14, 95% CI 0.03–0.76; *p* = 0.02), lower P-selectin levels (OR 0.11, 95% CI 0.02–0.56; *p* < 0.01), lower platelet counts (OR 0.52, 95% CI 0.30–0.90; *p* = 0.02) and vWF ratio < 0.7 (OR 8.64, 95% CI 1.47–50.80; *p* = 0.02) were associated with bleeding.

## Discussion

To our knowledge, this is the first study exploring the dynamics of thrombin generation by ST-Genesia together with other haemostatic variables along CART therapy, directed against CD19 and BCMA, and its potential association with haemostatic and immune complications. A progressive reduction in platelet count and P-selectin levels was observed during follow-up. CRP concentrations peaked markedly at day + 3, consistent with an acute inflammatory response after CART infusion and subsequently decreased below baseline levels by days + 14 and + 28. Although coagulation times showed some variability, ETP remained largely unchanged. However, fibrinogen decrease and D-dimer increase reflect some degree of persistent coagulation activation.

In our cohort, under routine LMWH prophylaxis and symptomatic VTE ascertainment, clinically relevant bleeding (13%) was more frequent than documented VTE (1.6%) within 30 days. Previous studies reported VTE rates up to 10% during CART therapy, while a recent meta-analysis described VTE and bleeding overall incidences of 2.4% and 1.9% per person-month, respectively [14]. Our findings, although the estimation of the incidence of thrombosis could not be completely precise, suggest that, at least during the first weeks after CART infusion, the risk of bleeding exceeds that of thrombosis, requiring particular attention. Although in our series thromboprophylaxis with LMWH during admission was widely used following current recommendations for cancer inpatients, only one patient maintained it at bleeding onset. To date, standardized strategies to prevent VTE and/or bleeding in this scenario are lacking.

A link between haemostasis and inflammation is confirmed by the association seen between ICANS and subsequent bleeding risk. This finding aligns with Johnsrud et al. [32], who reported a 50% incidence of bleeding complications among patients with ICANS. Indeed, endothelial dysfunction, platelet activation/consumption and coagulation impairment could be involved in the pathogenesis of both inflammatory and haemostatic adverse events [11, 14, 33]. Understanding these interactions is essential for developing effective interventions and improve clinical outcomes. Therefore, besides conventional coagulation tests and inflammatory biomarkers, we also evaluated TGA and P-selectin, given their potential role assessing hypo- and hypercoagulable states [19–21, 23, 34].

Baseline ETP and CRP were positively associated with increased risk of clinically relevant CRS and ICANS, respectively. Previous studies have focused on haemostatic abnormalities after CRS, typically between days 6 and 10 post-infusion, including prolonged coagulation times, hypofibrinogenemia, elevated D-dimer levels, and thrombocytopenia [35–37]. We also confirm some of these changes (i.e. increased D-dimer), though without predictive value. Regarding ICANS, the potential predictive role of CRP aligns with Gust et al. [38], although that study focused on peak levels during follow-up. In contrast, in our study no association between baseline fibrinogen and ICANS was found. Altogether, our findings suggest distinct haemostatic and inflammatory profiles for CRS and ICANS, which may aid clinicians in identifying patients requiring closer monitoring or early treatment with steroids or interleukin inhibitors.

Our results support the relevance of impaired coagulation and platelet function in CART-related bleeding [32, 39]. Platelet count appears as a consistent factor associated with the risk of bleeding during CART therapy given its association at baseline and subsequent time points. The cut-off of 100 × 10^9^/L suggested by the ROC curve analysis in the pre-lymphodepletion evaluation could imply changes in pharmacological thromboprophylaxis strategy in CART patients, moving up the usual threshold for withholding LMWH from 50 × 10^9^/L to 100 × 10^9^/L. Although mild thrombocytopenia is often undervalued by many clinicians, its impact in some clinical scenarios seems more relevant than expected, for example in the treatment of acute venous thromboembolism [40]. Moreover, baseline P-selectin levels were also negatively associated with the risk of bleeding. P-selectin is expressed in platelets and thrombin serves as a trigger for its release and activation [20]. Noteworthy, increased P-selectin has been associated with thrombosis, due to its role in cellular adhesion [20–23]. Whether this biomarker could guide decisions on thromboprophylaxis strategies or platelet transfusion thresholds in CART-recipient patients should be evaluated in specific studies.

Some predictive scores have been proposed to assess the risk of CART-related complications, such as the CAR-HEMATOTOX score for stratifying ICAHT risk [30, 31, 41]. In our cohort, baseline CAR-HEMATOTOX score was not associated with bleeding. Recently, the EASIX score, developed to evaluate endothelial damage in the setting of bone marrow transplant, has shown utility in predicting severe CRS and ICANS [29, 42, 43]. In our study, patients with bleeding had higher baseline EASIX scores. This score includes LDH, creatinine and platelet count [28, 42, 44]. Whether adding P-selectin could enhance its accuracy should be evaluated in larger cohorts.

There are some limitations to acknowledge. First, the relatively low number of patients, particularly with B-ALL, and events limits the statistical power and some of the evaluations (e.g. the associations with VTE risk or the number of covariates to include in multivariable analyses). However, our results support the role of the haemostatic system in the pathogenesis of CART complications and the potential use of some haemostatic tests as predictive biomarkers. Nevertheless, AUC values were modest and optimal cut-offs for decision-making should be refined in larger studies. Second, our study population comprised patients with B cell malignancies or multiple myeloma, treated with CD19- or BCMA-CARTs. Other products against different targets may behave differently. Third, patients requiring therapeutic anticoagulation, who may have higher thrombotic risk, were excluded due to the interference with most haemostatic tests. In this regard, some influence of the prophylactic-dose LMWH cannot be discarded. Finally, our study was conducted in two academic hospitals. Overall, given that the predictive performance and effect estimate of our results are uncertain, they should be considered hypothesis-generating. Multicentric confirmation of these exploratory results is necessary.

In conclusion, the risk of bleeding in the first month after BCMA or CD19-targeted CART infusion exceeds that of thrombosis. Our study provides valuable insights into the dynamics of the haemostatic profile of patients undergoing CART therapy. TGA, platelet count and P-selectin may help identify patients at high risk of developing CRS and haemorrhage allowing earlier targeted interventions.

## Supplementary Information

Below is the link to the electronic supplementary material.

Supplementary file1 (DOCX 281 KB)

## Data Availability

Data is available upon request.
