# Correction: Haemostatic changes during CART cell therapy and risk of complications

**DOI:** 10.1007/s00262-026-04425-9

**Published:** 2026-08-25

**Authors:** María Panizo-Inogés, María Marcos-Jubilar, Jose Ramón González-Porras, Carlos Puerta-Vazquez, Clara Fernández-Arias, Paula Rodríguez-Otero, Ana Alfonso‑Pierola, Sara Villar, Miguel Ángel Canales, Josune Orbe, Jose Antonio Páramo, Felipe Prósper, Ramón Lecumberri

**Affiliations:** 1 Hematology Department, Clinica Universidad de Navarra, Pamplona, Spain; 2 Hematology Department, Hospital Universitario de Salamanca, Salamanca, Spain

**Correction to: Cancer Immunology, Immunotherapy (2026) 75:130** 10.1007/s00262-026-04371-6

In the original version of this article, there are several formatting errors. Table 1 contains a systematic formatting error affecting multiple categorical variables, including “Sex”, “Diagnosis”, “Disease status pre-lymphodepletion”, and “Cardiovascular risk factors”. In these variables, the values of the first subcategory have been incorrectly placed in the header row (e.g., “Sex, n (%)”) instead of being aligned with their corresponding subcategory (e.g., “Male”). This results in a shift and misalignment of values across the first three columns, while the last column (*p*-values) appears correctly positioned and Table 2 has also been incorrectly formatted due to a column shift during typesetting. The column containing parameter names (e.g., “Lag time (ratio)”, “Peak Height (%)”) has been displaced into the column corresponding to baseline values. Consequently, all subsequent cells are misaligned.

The correct Tables 1 and 2 should have appeared as shown below.

**Table 1:** Baseline characteristics of the recruited patients

	Total (n = 62)	Anti-CD19 CART (n = 33)	Anti-BCMA CART (n = 29)	*p* value
Age (years) (median, IQR)	62 (53–69)	62 (53–68)	65 (53–69)	0.54
Sex, n (%)				0.36
Male	40 (65)	23 (70)	17 (59)	
Female	22 (35)	10 (30)	12 (41)	
Diagnosis, n (%)				NA
B-ALL	2 (3)	2 (6)	NA	
B-cell lymphomas	31 (50)	31 (94)	NA	
Multiple myeloma	29 (47)	NA	29 (100)	
Disease status pre-lymphodepletion, n (%)				0.07
CR	7 (11)	2 (6)	5 (17)	
PR	14 (23)	5 (15)	9 (31)	
NR/PD	41 (66)	26 (79)	15 (52)	
History of venous thrombosis, n (%)	15 (24)	8 (24)	7 (24)	0.99
PE ± DVT	7 (12)	2 (6)	5 (17)	
Isolated lower-limb DVT	4 (6)	4 (12)	0 (0)	
CRT	4 (6)	2 (6)	2 (7)	
History of arterial thrombosis, n (%)	5 (8)	4 (12)	1 (3)	0.61
Myocardial infarction	2 (3)	2 (6)	0 (0)	
Stroke	3 (5)	2 (6)	1 (3)	
Cardiovascular risk factors, n (%)				
HT	20 (32)	11 (33)	9 (31)	0.85
DM	10 (16)	9 (27)	1 (3)	0.01
DL	16 (25)	10 (30)	6 (21)	0.39
CKD	7 (7)	6 (18)	1 (3)	0.11
Thromboprophylaxis upon admission, n (%)	51 (82)	25 (75)	26 (90)	0.22
Duration (days) (median, IQR)	14 (12–15)	14 (10–18)	14 (13–14)	
CAR-HEMATOTOX ≥ 2 points, n (%)	24 (39)	16 (48)	8 (28)	0.07
EASIX score (median, IQR)	1.21 (0.78–2.59)	1.52 (1.03–2.59)	1.04 (0.69–2.47)	0.07

B-ALL: B-cell acute lymphoblastic leukaemia. CR: complete response. PR: partial response. NR: no response. PD: progressive disease. PE ± DVT: pulmonary embolism ± deep vein thrombosis. CRT: catheter related thrombosis. HT: hypertension. DM: diabetes mellitus. DL: dyslipidaemia. CKD: chronic kidney disease. IQR: interquartile range. NA: Not applicable

**Table 2:** Dynamics of thrombin generation variables and other haemostatic tests

		Baseline	Pre-Infusion	Day + 3	Day + 14	Day + 28
Thrombin Generation	Lag time (ratio)	1.3 (1.1–1.5)	1.4 (1.2–1.9) *	1.3 (1.2–1.8) *	1.2 (1.0–1.5) †	1.2 (1–1.5) †
	Peak Height (%)	107.3 (73.1–137.0)	80.6 (57.2–108.4) *	90.5 (61.5–118.3) *	97.5 (75.3–122.4) †	91.1 (67.8–115.7) †
	Time to Peak (ratio)	1.2 (0.9–1.4)	1.3 (1.1–1.6) *	1.3 (1.1–1.6) *	1.2 (1.1–1.4) †	1.2 (1.1–1.6) †
	ETP (%)	99.3 (78.9–106.5)	92.7 (72.4–109.1)	97.4 (80.3–111.1)	94.1 (81.5–107.7)	94.8 (77.5–106.6)
	TM ETP Inh (%)	47.4 (26.7–62.2)	51.1 (39.1–64.6)	48.6 (32.8–57.1)	50.1 (37.6–61.7)	52.1 (33.0–72.9)
Prothrombin Time (s) (RV: 9.2–13.8)		9.7 (8.4–13.9)	12.2 (8.2–14.1)	11.5 (8.4–14.6) * †	11.2 (9.3–14.4) * †	11.4 (8.9–13.4)*†
Activated Partial Thromboplastin Time (s) (RV: 24.5–35.5)		30.5 (27.9–33.2)	31.1 (28.2–34.6)	33.8 (28.5–37.4) * †	27.5 (24.3–31.3) * †	26.6 (24.2–30.3)* †
D-dimer (ng/mL) (RV: < 500)		570 (300–930)	700 (440–1500) *	900 (5001500) *	900 (570–1940) * †	702 (400–1455)
Fibrinogen (mg/dL) (RV: 200–393)		390 (328–478)	409 (344–463)	415 (360–473)	182 (142–227) * †	182 (152–242)*†
vWF Act/Ag (ratio) (RV ≥ 0.7)		0.9 (0.8–1.1)	0.9 (0.8–1.1)	0.9 (0.8–1.1)	0.8 (0.7–0.9) * †	0.8 (0.6–0.9) *†
CRP (mg/dL) (RV ≤ 0.5)		0.6 (0.1–1.8)	0.7 (0.4–2.3)	2.2 (0.5–5.9) * †	0.2 (0.1–0.4) * †	0.1 (0.04–0.1)*†
Platelets (× 10^9^/L) (RV: 150–450)		156 (106–201)	125 (66–161) *	101 (51–145) * †	101 (31–152) * †	65 (16–112) * †
P-selectin (ng/ml)		46.4 (38.8–55.3)	31.6 (25.9–38.4)*	30.0 (23.3- 39.8)* †	40.5 (27.7–50.3)* †	NA

Available reference values are provided where applicable. All data are presented as median (interquartile range). *p < 0.05 compared to pre-lymphodepletion. †*p* < 0.05 compared to pre-infusion. ETP: endogenous thrombin potential. TM: thrombomodulin. vWF Act/Ag: von Willebrand factor activity/antigen ratio. CRP: C-reactive protein. RV: reference values. NA: not available. At day + 28, 18 cases were missing due to losses to follow-up

The original article has been corrected.

