# Supplementary material for: Haemostatic changes during CART cell therapy and risk of complications
Source: Cancer Immunol Immunother. 2026 Mar 31;75(4):130. doi: 10.1007/s00262-026-04371-6 (PMC13038757; doi:10.1007/s00262-026-04371-6)
Supplement: Supplementary file 1 — Supplementary file1 (DOCX 281 KB) [file 262_2026_4371_MOESM1_ESM.docx]

**
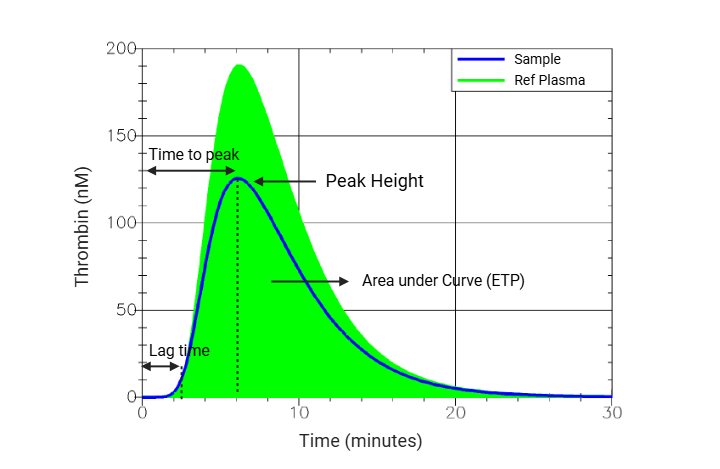
**

**Supplementary Figure 1** Representation of the thrombin generation assay (TGA) performed with the ST-Genesia system. The thrombin generation curve illustrates the main parameters derived from the assay: lag time (time until initiation of thrombin generation), time to peak (time to reach maximum thrombin concentration), peak height (maximum thrombin concentration generated), and endogenous thrombin potential (ETP, area under the thrombin generation curve), reflecting the overall capacity to generate thrombin.

**Supplementary Table 1** Bleeding and Thrombotic Events during follow-up.

|  | Bleeding  nº1 | Bleeding  nº2 | Bleeding  nº3 | Bleeding  nº4 | Bleeding  nº5 | Bleeding  nº6 | Bleeding  nº7 | Bleeding  nº8 | Thrombosis |
| --- | --- | --- | --- | --- | --- | --- | --- | --- | --- |
| Diagnosis | BCL | BCL | BCL | MM | BCL | BCL | MM | BCL | BCL |
| Location | GI | GI | GI | CNS | Urinary | Muscle hematoma | GI | CNS | CRT |
| Day of Onset | 16 | 30 | 20 | 27 | 28 | 14 | 28 | 10 | 20 |
| Bleeding Severity (ISTH) | Major | Major | Major | Major | Non-major, but clinically relevant | Non-major, but clinically relevant | Major | Major | NA |
| Thromboprophylaxis During Hospitalization | None | LMWH | None | None | None | LMWH | LMWH | None | LMWH |
| On thromboprophylaxis at Day +3 | No | Yes | No | No | No | Yes | No | No | Yes |
| On thromboprophylaxis at Time of Event | No | No | No | No | No | Yes | No | No | No |
| Baseline Platelet count (x10^9^/L) | 23 | 81 | 10 | 10 | 288 | 251 | 98 | 46 | 109 |
| Platelet count at day +3 (x10^9^/L) | 8 | 75 | 8 | 9 | 133 | 95 | 67 | 20 | 91 |
| CRS grade | 1 | 2 | 2 | 1 | 1 | 1 | 1 | 2 | 2 |
| ICANS grade | 1 | 4 | 4 | No ICANS | No ICANS | 1 | 2 | 3 | No ICANS |

BCL: B-cell lymphoma. MM: multiple myeloma. GI: gastrointestinal. CNS: central nervous system. CRT: catheter related thrombosis. ISTH: International Society on Thrombosis and Haemostasis. CRS: cytokine release syndrome. ICANS: immune effector cell-associated neurotoxicity syndrome. NA: not applicable.


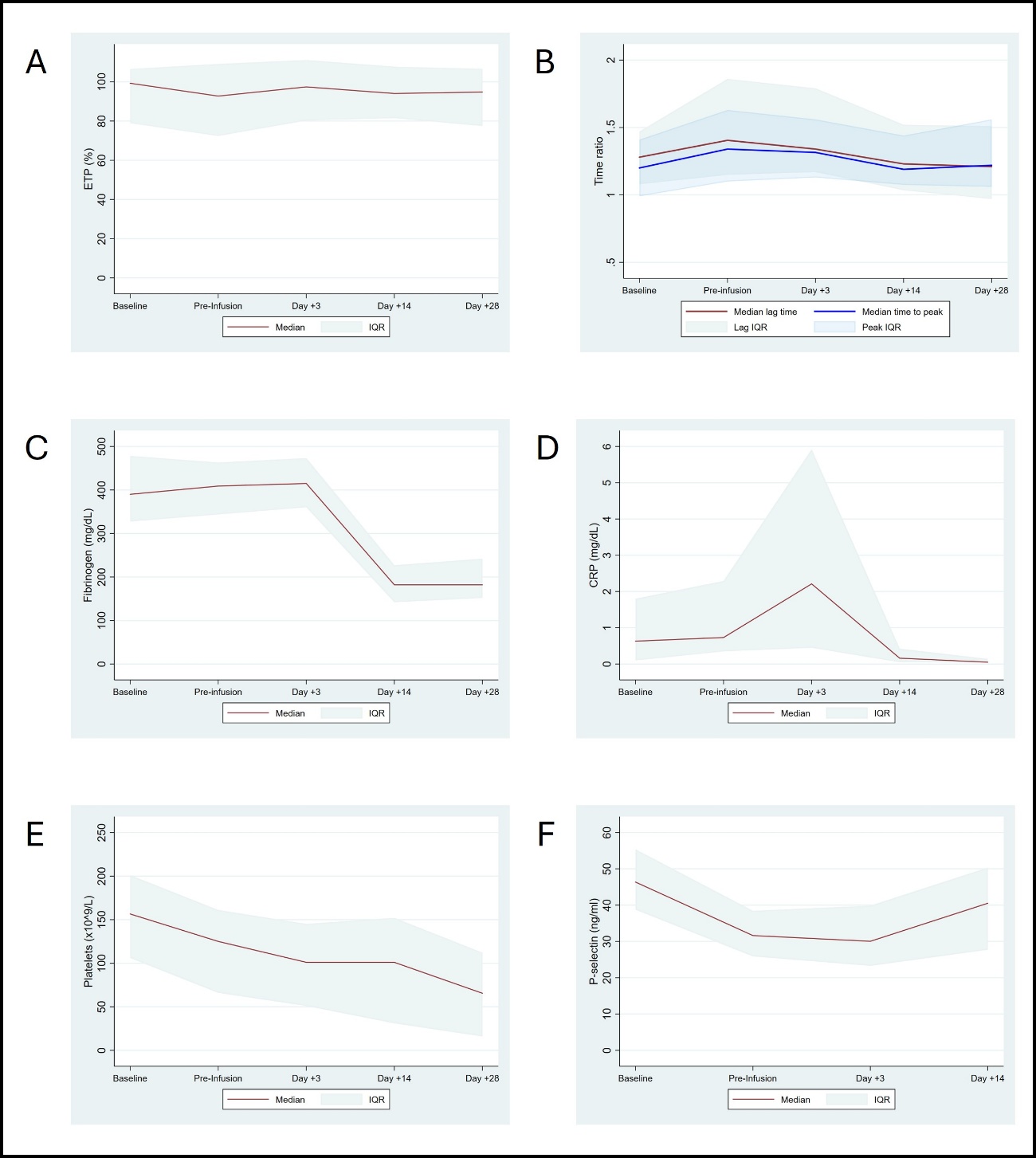


**Supplementary Figure 2** Dynamics of key thrombin generation variables and biomarkers. Panels show the temporal evolution of (a) endogenous thrombin potential (ETP), (b) lag time and time to peak, (c) fibrinogen, (d) C-reactive protein (CRP), (e) platelet count, and (f) P-selectin. Data are presented as median with interquartile range.
